# Supplementary material for: Evidence for the Effectiveness of Psychological Interventions for Internalized Stigma among Adults with Schizophrenia Spectrum Disorders: A Systematic Review and Meta-Analyses
Source: Int J Environ Res Public Health. 2023 Apr 18;20(8):5570. doi: 10.3390/ijerph20085570 (PMC10138403; doi:10.3390/ijerph20085570)
Supplement: Supplementary file 1 [file ijerph-20-05570-s001.zip › Table S2.pdf]

Table S2. Summary of the NHRMC Evidence Statement Matrix

**NHMRC BODY OF EVIDENCE MATRIX**		
1.Evidence base	A - excellent	18/27 are level II studies; 16/18 with low risk of bias
2.Consistency	B - good	most studies consistent and inconsistency may be explained
3.Clinical impact	A - excellent	very large potential benefit from application of the guideline to a population
4.Generalizability	B - good	The included studies were conducted in different countries. However, the evidence is sensible to be generalized to all adults with schizophrenia spectrum disorder
5. Applicability	B - good	applicable to local healthcare context with few caveats
**Grade of recommendation**	**B - good**	**Body of evidence provides some support for recommendation(s) but care should be taken in its application**
